# Supplementary material for: Dredging and dumping impact coastal fluxes of sediment and organic carbon
Source: Nat Commun. 2026 Jan 7;17:216. doi: 10.1038/s41467-025-68105-5 (PMC12780194; doi:10.1038/s41467-025-68105-5)
Supplement: Supplementary file 1 — Supplementary Information [file 41467_2025_68105_MOESM1_ESM.pdf]

Supplement to: Dredging and dumping impact coastal fluxes of sediment and organic carbon

Lucas Porz[1,*], Jiayue Chen[1], Ruemeysa Yilmaz[1], Jannis Kuhlmann[2], Wenyan Zhang[1,*], Corinna Schrum[1,3]

[1]Institute of Coastal Systems, Helmholtz-Zentrum Hereon, Max-Planck-Strasse 1, 21502 Geesthacht, Germany

[2]BUND-Meeresschutzbüro, Bund für Umwelt und Naturschutz Deutschland e.V. (BUND), Bremen, Germany

[3]Institute of Oceanography, Center for Earth System Research and Sustainability, Universität Hamburg, Bundesstrasse 53, 20146 Hamburg, Germany

*Correspondence to: Lucas Porz (lucas.porz@hereon.de), Wenyan Zhang (wenyan.zhang@hereon.de)

Supplementary Table 1. Dumped sediment type classification. Classifications are assigned to dumping activities according to description strings in the dumping data[1]. Activities with missing or non-attributable sediment type descriptions were categorized as mixed. The classification is used to assign OC contents in dumped material of 2–8% (average: 5%) and 0–2% (average: 1%) in the fine and coarse fractions, respectively. Mixed material was assumed to comprise coarse and fine sediment in equal parts.

	Classification		
	Fine-grained	Coarse-grained	Mixed
Identifiers	'SILT'	'Gravels'	'Gravel/Silt'
	'Silt'	'SAND'	'Gravels / Silts'
	'Silts'	'Sand'	'SAND/SILT'
	'clay'	'Sands'	'Sand and silt'
	'clay and silt'	'Sands / Gravels'	'Sand/Silt'
	'clay, till'	'gravel'	'Sands / Silts'
	'cohesive soil'	'gravel/sand'	'Silt and Sand'
	'glacial clay'	'mainly sand'	'Silt/Sand'
	'glacial clay, clay'	'non-cohesive soil'	'Silt/sand'
	'mainly silt'	'predom. sand'	'mix'
	'mud'	'rocks'	'mix of silt and sand'
	'postglacial clay'	'sand'	'mud/sand'
	'silt'	'sand and gravel'	'sand / silt'
		'sand/gravel'	'sand /silt mixed'
			'sand and silt'
			'sand- silt'
			'sand/merl'
			'sand/silt'
			'sand/silt mixed'
			'sand/silt/gravel'
			'silt , finesand'
			'silt, fine sand'
			'silt/sand'
			'silt/sand/clay'
			'various'

Supplementary Methods 1: Material extraction model implementation

The material extraction simulations use the hydrodynamic SCHISM model setup of Kossack et al.[2] coupled to the model TOCMAIM[3], which uses 30 dynamic sediment layers and 6 sediment classes assigned according to sediment maps[4] (with sediment dynamic properties set as in Porz et al. (2024)[5] listed in Table S1). The natural organic carbon degradation is reduced exponentially below the oxygen penetration depth within the sediment to represent reduced mineralisation with lower oxygen availability. Oxygen penetration depends on sediment bed grain size, with sandy sediments having higher oxygen penetration than muddy sediments.

The average extracted mass between 1992 and 2023 of 104 Mt yr^{-1} is divided into a total extraction area of 3.575 km², which equates to a bulk sediment disturbance of 0.92 mg m^{-2} s^{-1} for the continuous extraction scenario and 6.44 mg m^{-2} s^{-1} for the weekly extraction scenario. This disturbance is divided among the sediment classes in the seabed according to their composition, with the sand component being removed and the silt, clay and OC classes being resuspended into the water column, where they may re-deposit, be mixed upward by turbulence, and transported with the current, depending on the hydrodynamic conditions. The resulting change in sediment OC is shown in Supplementary Figure 1.

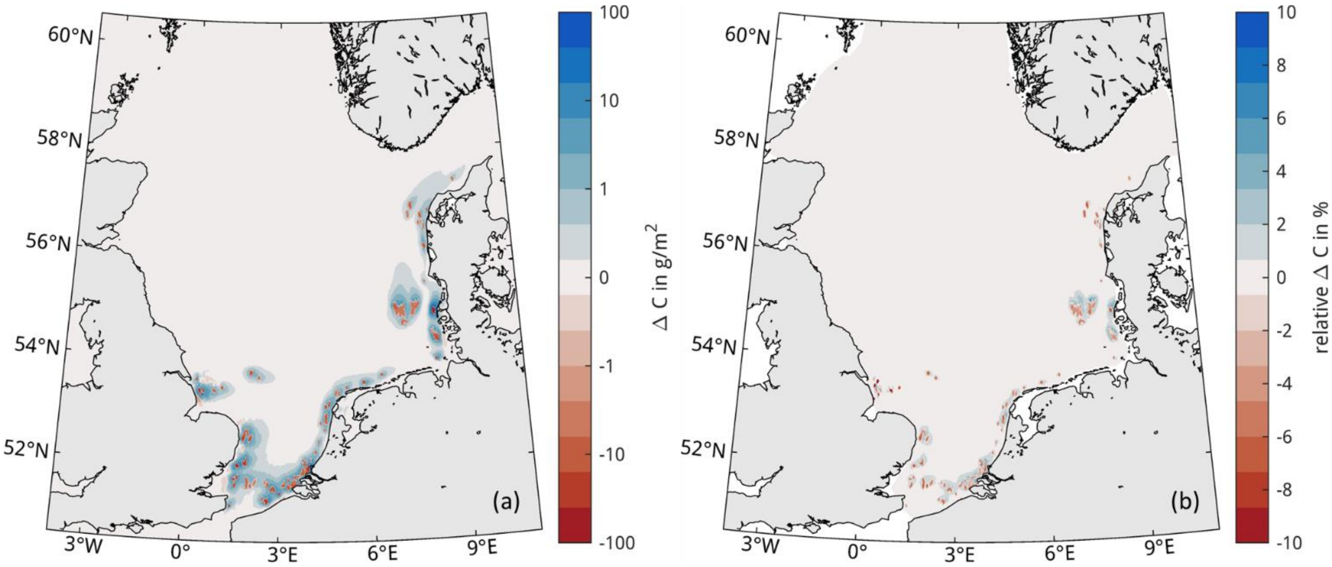

Supplementary Figure 1. Material extraction simulation results. (a) Absolute and (b) relative change in sediment organic carbon distribution after one year of continuous sediment extraction within the extraction sites. Negative values (red) indicate loss of carbon compared to the reference simulation without extraction.

Supplementary Table 2. Material extraction sediment parameter settings. For each fraction, set values are given for settling velocity (w_s), critical shear stress for resuspension (τ_c), erosion rate (M_E; erosion formulation according to Winterwerp et al.[6]) and remineralization rate (r).

	Inorganic (inert)			Organic		
	Clay	Silt	Sand	High reactivity	Medium reactivity	Low reactivity
w_s (mm s^{-1})	0.005	0.1	0.2		0.1	
τ_c (Pa)	0.1	0.1	0.2		0.1	
M_E (s m^{-1})	0.001	0.001	0.002		0.001	
r (d^{-1})	-	-	-	$5.5\cdot10^{-2}$	$5.5\cdot10^{-3}$	$5.5\cdot10^{-5}$

Supplementary Methods 2: Dumping model implementation

The dumping simulations use the hydrodynamic SCHISM model setup of Chen et al. (2025)[7], which has been validated for hydrodynamics and sediment dynamics in the German Bight and Wadden Sea. For the dumping experiments, the seafloor is left bare, i.e., no sediment is initialized in the model, as we are only interested in the redistribution of dumped material. However, the heterogeneity of the seafloor is accounted for in this setup through consideration of grain-size dependant seabed roughness, that is, coarse-grained seabed has a larger bottom drag coefficient compared to fine-grained seabed.

Based on the estimated dumped OC amounts assuming an average OC content of 5% in fine-grained and 1% in coarse-grained material, representing the midpoints of the estimated ranges, dumping was implemented as an additional constant source of water and fine-grained sediment with dynamic properties (listed in Supplementary Table 2) at the sea surface at each model grid cell nearest to each dumping location. For each location, dumped volume rates (in m³ s^{-1}) were assigned as

$$v = \frac{m_{\mathrm{dump,OC}}}{C}, \tag{1}$$

where $m_{\mathrm{dump,OC}}$ (in kg s^{-1}) is the average OC dumping rate according to our estimate, and C (in kg m^{-3}) is the corresponding sediment mass concentration of the mixture. The solid and water volumes of the mixture can be expressed as $V_S = M_S/\rho_S$ and $V_W = WC \cdot M_S/\rho_W$, respectively, where $\rho_W = 1{,}000\ \mathrm{kg\ m^{-3}}$ is the water density, $\rho_S = 2650\ \mathrm{kg\ m^{-3}}$ is the average sediment grain density, and $WC =$

$M_W/M_S = 300\%$ is the water content of bulk sediment (expressed as weight water M_W per weight dry bulk sediment M_S) based on measurements in estuarine muds[8]. The total volume of the mixture is then $V = V_S + V_W = M_S \cdot \left(1/\rho_S + WC/\rho_W\right)$. Finally, the mass concentration of solids in the dumped water volume is calculated to the constant value of

$$C = \frac{M_S}{V} = \frac{\rho_W \cdot \rho_S}{\rho_W + \rho_S \cdot WC} = 296\,\frac{\text{kg}}{\text{m}^3}, \quad (2)$$

It is noteworthy that the value of WC used here may be an overestimate, since dredged mud is typically drained in the hoppers, leading to considerably lower dumped water volumes. However, the volume of water added with dumping does not affect the model results significantly, as it is negligible compared to the model grid cell volume.

Supplementary Table 3. Dumping sediment parameter settings. Set values are given for median grain size (D_{50}), range of feasible settling velocities (w_s) for the study area[9], critical shear stress for resuspension (τ_c), and erosion rate (M_E).

Parameter	Value
D_{50} (µm)	20
w_s (mm s^{-1})	0.05 or 1
τ_c (Pa)	0.1
M_E (s m^{-1})	0.001

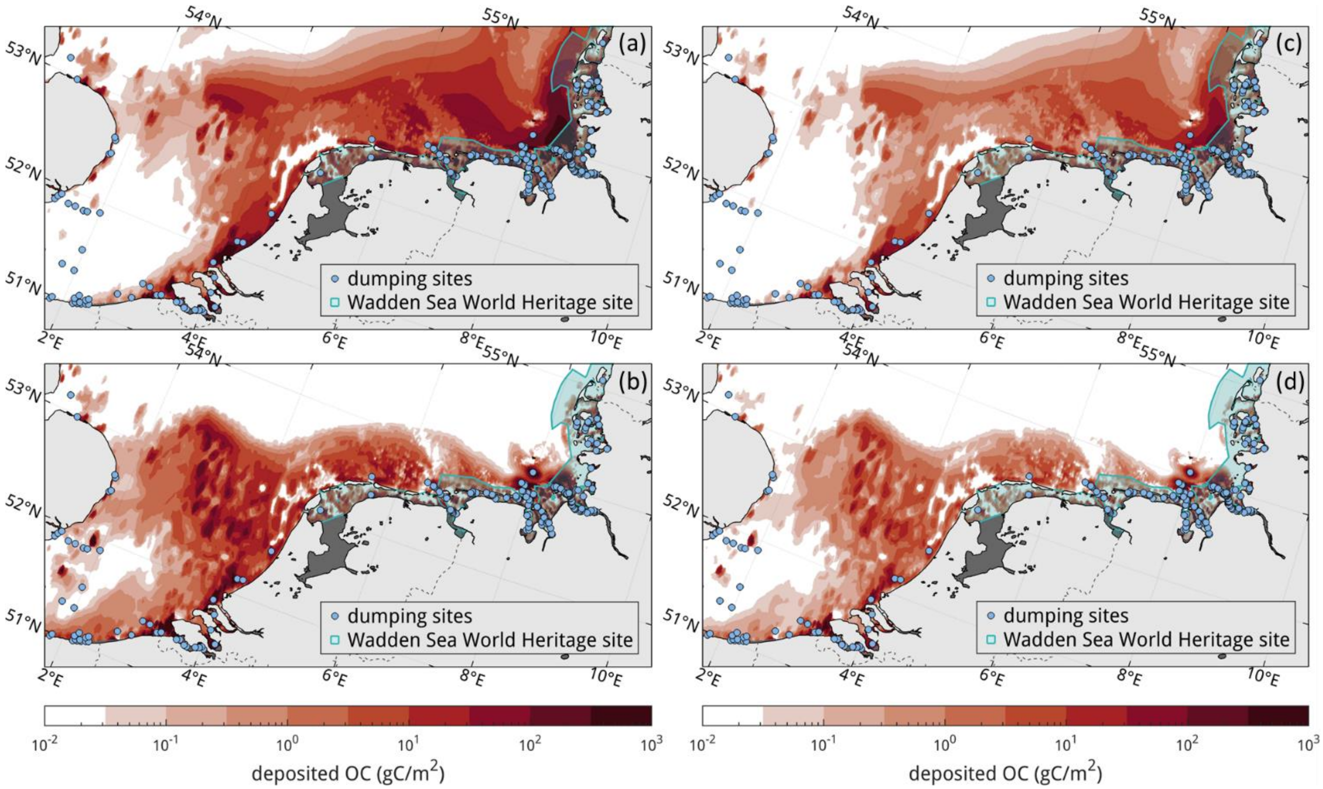

Supplementary Figure 2. Ranges of dumping simulation results. Spatial distribution of dumped sediment organic carbon in the southeastern North Sea area after one year of continuous dumping with particle sinking velocities of (a,c) 0.05 mm s^{-1} and (b,d) 1 mm s^{-1} and with organic carbon concentrations in the dumped material of (a,b) 8% (fine fraction) and 2% (coarse fraction), and (c,d) 2% (fine fraction) and 0% (coarse fraction) are shown. Note the logarithmic colour mapping. Dumping locations are marked as blue circles and Wadden Sea World Heritage site in blue shading. Areas in dark grey are not included in the model domain. Projection: oblique Mercator.

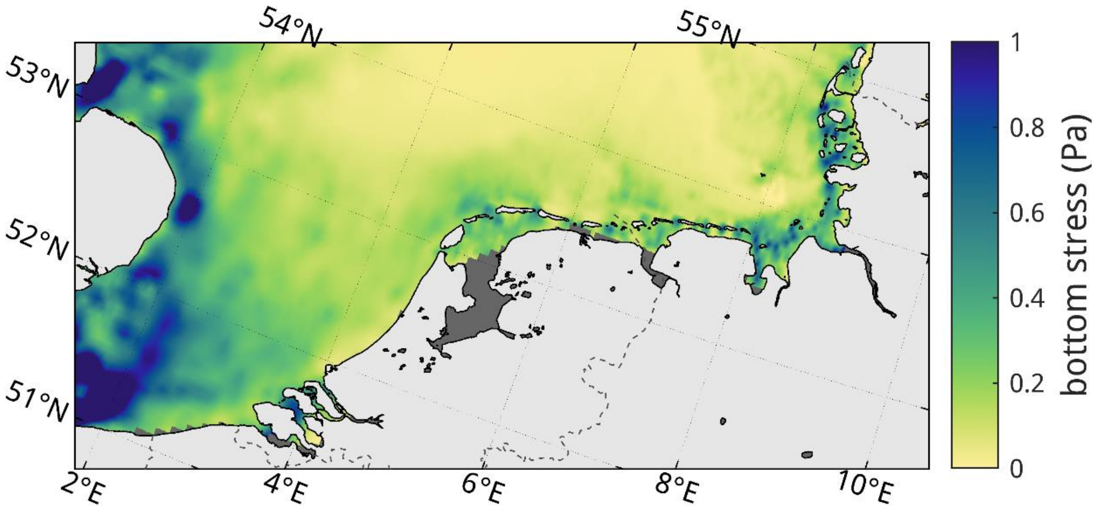

Supplementary Figure 3. Simulated bottom shear stress. Values are averaged from hourly outputs for the last day of the hydrodynamic simulation (Dec 30th, 2014).

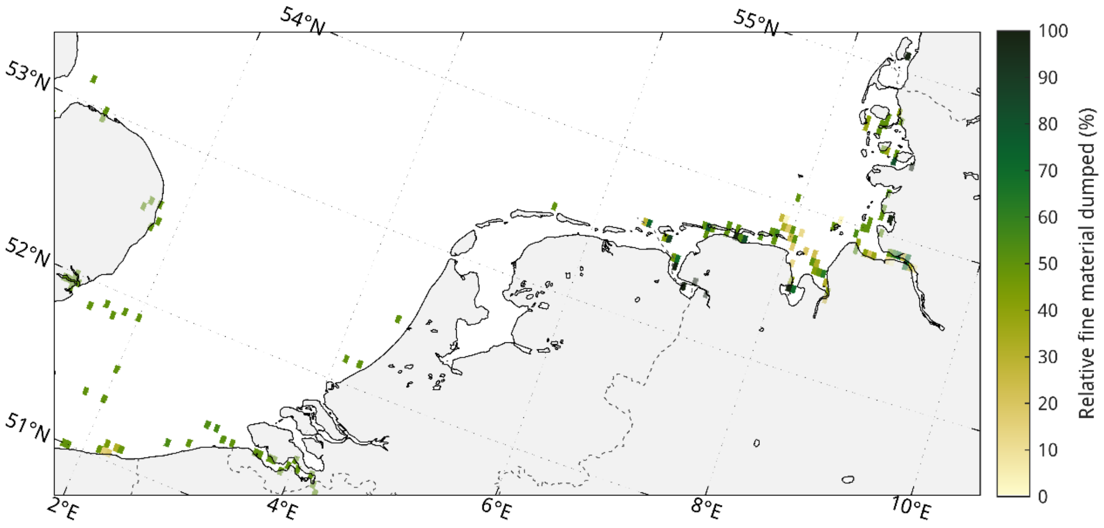

Supplementary Figure 4. Relative proportions of fine-grained sediment in dumped material. Values are averaged for 1995 to 2021 and cumulated on a 3’×3’- grid. The amount of fine-grained material largely determines the estimated amount of organic carbon added in the model.

References

1. OSPAR Commission (OSPAR). Dumping and Placement of Wastes or Other Matter at Sea. Data Source: OSPAR Data and Information Management System. Available at https://odims.ospar.org/en/submissions/ospar_dumping_at_sea_2021_01/ (2021).
2. Kossack, J., Mathis, M., Daewel, U., Zhang, Y. J. & Schrum, C. Barotropic and baroclinic tides increase primary production on the Northwest European Shelf. *Front. Mar. Sci.* **10**; 10.3389/fmars.2023.1206062 (2023).
3. Zhang, W. *et al.* The Budget of Macrobenthic Reworked Organic Carbon: A Modeling Case Study of the North Sea. *J. Geophys. Res. Biogeosci.* **124,** 1446–1471; 10.1029/2019JG005109 (2019).
4. Bockelmann, F.-D., Puls, W., Kleeberg, U., Müller, D. & Emeis, K.-C. Mapping mud content and median grain-size of North Sea sediments – A geostatistical approach. *Mar. Geol.* **397,** 60–71; 10.1016/j.margeo.2017.11.003 (2018).
5. Porz, L. *et al.* Quantification and mitigation of bottom-trawling impacts on sedimentary organic carbon stocks in the North Sea. *Biogeosciences* **21,** 2547–2570; 10.5194/bg-21-2547-2024 (2024).
6. Winterwerp, J. C., van Kesteren, W. G. M., van Prooijen, B. & Jacobs, W. A conceptual framework for shear flow–induced erosion of soft cohesive sediment beds. *J. Geophys. Res. Oceans* **117**; 10.1029/2012JC008072 (2012).
7. Chen, J. *et al.* Physical Mechanisms of Sediment Trapping and Deposition on Spatially Confined Mud Depocenters in High-Energy Shelf Seas. *J. Geophys. Res. Oceans* **130,** e2025JC022622; 10.1029/2025JC022622 (2025).
8. Zander, F., Heimovaara, T. & Gebert, J. Spatial variability of organic matter degradability in tidal Elbe sediments. *Journal of Soils and Sediments* **20,** 2573–2587; 10.1007/s11368-020-02569-4 (2020).
9. Maerz, J. *et al.* Maximum sinking velocities of suspended particulate matter in a coastal transition zone. *Biogeosciences* **13,** 4863–4876; 10.5194/bg-13-4863-2016 (2016).
